# Cocoa and Grape Seed Byproducts as a Source of Antioxidant and Anti-Inflammatory Proanthocyanidins

**DOI:** 10.3390/ijms18020376

**Published:** 2017-02-10

**Authors:** María De La Luz Cádiz-Gurrea, Isabel Borrás-Linares, Jesús Lozano-Sánchez, Jorge Joven, Salvador Fernández-Arroyo, Antonio Segura-Carretero

**Affiliations:** 1 Department of Analytical Chemistry, University of Granada, c/Fuentenueva s/n, 18071 Granada, Spain; mluzcadiz@ugr.es (M.D.L.L.C.-G.); iborras@ugr.es (I.B.-L.); jesusls@ugr.es (J.L.-S.); ansegura@ugr.es (A.S.-C.); 2 Research and Development of Functional Food Centre (CIDAF), PTS Granada, Avda. Del Conocimiento s/n, Edificio BioRegion, 18016 Granada, Spain; 3 Centre de Recerca Biomèdica, Institut d’Investigació Sanitària Pere Virgili, Hospital Universitari de Sant Joan, Universitat Rovira i Virgili, C/Sant Joan s/n, 43201 Reus, Spain; jorge.joven@urv.cat

**Keywords:** *Vitis vinifera* seed, byproduct, *Theobroma cacao*, HPLC-ESI-QTOF-MS, polyphenols, proanthocyandins, antioxidant activity, anti-inflammatory activity

## Abstract

Phenolic compounds, which are secondary plant metabolites, are considered an integral part of the human diet. Physiological properties of dietary polyphenols have come to the attention in recent years. Especially, proanthocyanidins (ranging from dimers to decamers) have demonstrated potential interactions with biological systems, such as antiviral, antibacterial, molluscicidal, enzyme-inhibiting, antioxidant, and radical-scavenging properties. Agroindustry produces a considerable amount of phenolic-rich sources, and the ability of polyphenolic structures to interacts with other molecules in living organisms confers their beneficial properties. Cocoa wastes and grape seeds and skin byproducts are a source of several phenolic compounds, particularly mono-, oligo-, and polymeric proanthocyanidins. The aim of this work is to compare the phenolic composition of *Theobroma cacao* and *Vitis vinifera* grape seed extracts by high pressure liquid chromatography coupled to a quadrupole time-of-flight mass spectrometer and equipped with an electrospray ionization interface (HPLC-ESI-QTOF-MS) and its phenolic quantitation in order to evaluate the proanthocyanidin profile. The antioxidant capacity was measured by different methods, including electron transfer and hydrogen atom transfer-based mechanisms, and total phenolic and flavan-3-ol contents were carried out by Folin–Ciocalteu and Vanillin assays. In addition, to assess the anti-inflammatory capacity, the expression of MCP-1 in human umbilical vein endothelial cells was measured.

## 1. Introduction

Phenolic compounds are ubiquitous compounds found in most fruits and vegetables and are associated with multiple biological activities, including radioprotective, anti-inflammatory, anti-carcinogenic, antiviral and antibacterial properties attributed mainly to their antioxidant and antiradical activity [1,2]. Increasing the antioxidant compounds intake in the human diet and, for example, enriching food with antioxidant compounds, is considered important. As some synthetic antioxidants may exhibit toxicity and require high manufacturing costs showing lower efficiency than natural antioxidants, there is a need to identify natural and possibly more economic and effective antioxidants with potential to be incorporated into foods [3]. Several studies show the effect of bioactive compounds on gene expression and its impact on metabolic pathways to prevent and/or ameliorate symptoms in some diseases [4] and, therefore, its rational use is an open door in alternate medicine or pharmaceutical industry.

Tannins (comprising hydrolysable and condensed tannins) are one of the major groups of polyphenols which are found in our diets. Proanthocyanidins (belonging condensed tannins) have been identified in several agricultural byproducts, seeds, fruits and vegetables [5] and their biological metabolism and pharmacokinetics have been extensively reviewed [6]. In addition to the free radical scavenging and antioxidant activity [7], proanthocyanidins also exhibit vasodilatory, anti-carcinogenic, anti-allergic, anti-inflammatory, anti-bacterial, cardio-protective, immune-stimulating, anti-viral and estrogenic activities [8], as well as are inhibitors of the enzymes phospholipase A2 [9], cyclooxygenase and lipooxygenase [10]. The anti-inflammatory activity of proanthocyanidins is one of the most widely studied [11,12,13,14,15,16,17]. Mechanisms of action include modulation of the arachidonic acid and nuclear factor-κB (NF-κB) pathways, inhibition of eicosanoid generating enzymes, inflammatory mediator secretion and the mitogen-activated protein kinase pathway [18].

**Figure 1:**
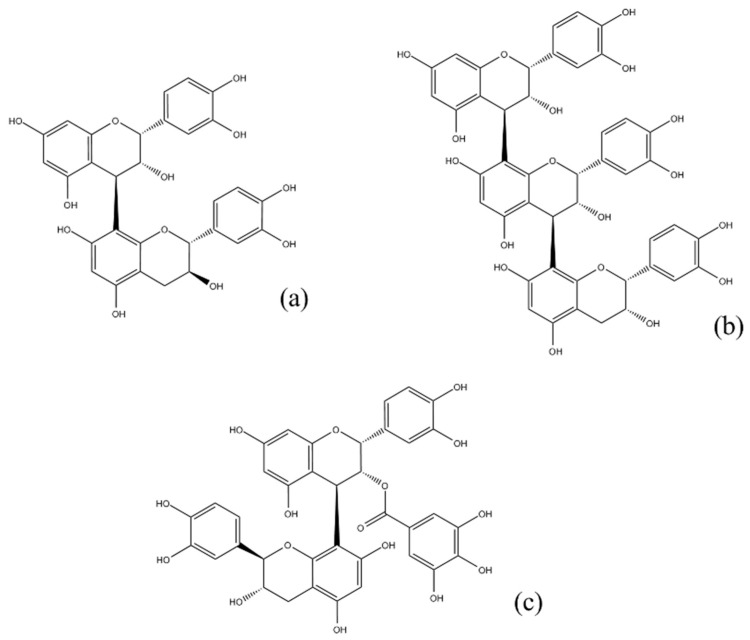
Most common proanthocyanidins in cacao and grape seed extracts: procyanidin dimer (**a**); procyanidin trimer (**b**); and procyanidin dimer gallate (**c**).

Grapes (*Vitis vinifera* L.) are one of the most widely grown fruit crops throughout the world, and their composition and properties have been extensively investigated, with several reports of the presence of phenolic compounds [19,20]. Grape seeds, amounting to 38%–52% on a dry matter basis [21], are a considerable proportion of the industrial byproduct from the winemaking process. Grape seeds constitute a cheap source of antioxidant compounds due to their incomplete extraction during wine production, providing important economic advantages [20,22]. Cocoa and cocoa products, i.e. cocoa liquor, cocoa powder and chocolates, are worldwide consumed and common ingredients of many food products. The chocolate market has remained stable during the last years [23] and, alternatively, the scientific interest on this potential bioactive source has grown at exponential levels. In grape seeds and cocoa extracts, proanthocyanidins represent the major part of the total polyphenolic extract. These compounds are, in fact, composed of chains of flavan-3-ols units, (+)-catechin and (−)-epicatechin, linked together through C4–C6 and C4–C8 interflavanoid bonds, and various gallate esters [24] (Figure 1).

Therefore, the main objectives of this work were: (1) to investigate and improve the knowledge of the composition profile at the present time [25], mainly proanthocyanidins, in a grape seed extract using high pressure liquid chromatography (HPLC) coupled to a quadrupole time-of-flight mass spectrometer (QTOF-MS) and equipped with an electrospray ionization (ESI) interface; (2) to evaluate the total phenolic and flavan-3-ol content by Folin–Ciocalteu and Vanillin assays, respectively; (3) to known how proanthocyanidins operate by determination and comparison of the antioxidant potential in vitro; and (4) to know the anti-inflammatory potential of grape seed and cocoa extracts measuring the expression of MCP-1 in human umbilical vein endothelial cells (HUVECs) [26]. These aims are a first approach in order to find bioactive compounds with biological properties that could be used as preventive or treatment of different pathophysiological disorders.

## 2. Results and Discussion

### 2.1. Characterization of Grape Seed Extract by HPLC-ESI-QTOF-MS

A comprehensive analytical characterization of phenolic compounds using advanced and powerful techniques is crucial. In this way, suitable methods need to be established for the characterization of bioactive compounds in vegetable matrices. The interpretation was performed based on both exact mass and tandem mass spectra allowed by the QTOF technologies, which is essential for elemental composition assignment and, thus, for the characterization of small molecules. In the next sections, we do not consider decimals in exact mass and fragments to a better understanding of the manuscript. For exact masses including decimals, refer to Table 1.

**Figure 2:**
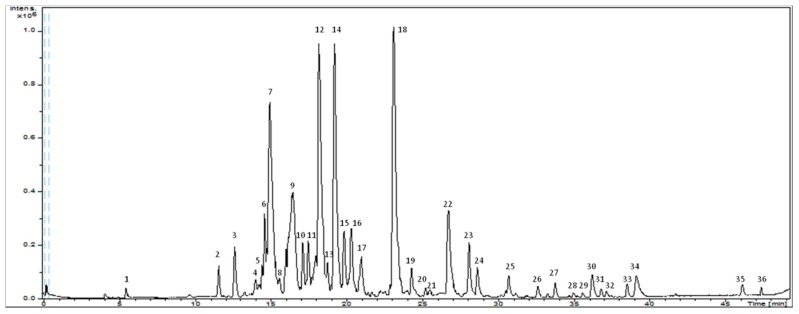
Base peak chromatogram of grape seed extract.

A total of 36 compounds distributed in three major categories have been analyzed in grape seeds extract: (1) phenolic acids; (2) flavonoids (flavan-3-ol, procyanidins and others flavonoids); and (3) other compounds. Note that a comprehensive characterization of cocoa extract has already been published by Cádiz-Gurrea et al. (2014) [27]. Figure 2 shows the base peak chromatogram (BPC) of the grape seed extract and the major peaks observed has been assigned in Table 1, identified considering the elution order. All the compounds were characterized by the interpretation of their mass spectra obtained by the QTOF-MS and also taking into account the data provided by the literature, as explained in the following sections.

#### 2.1.1. Phenolic Acids

Peak **2**, identified as gallic acid at *m/z* 169 yield a fragment at *m/z* 125 due to the decarboxylation of the galloyl group [20,28] and confirmed by comparison with the retention time of the standard. Peak **34** yield a [M − H]^−^ at *m*/*z* 301 and was tentatively characterized as ellagic acid [29].

#### 2.1.2. Flavonoids

Peaks **12**, **18**, **22** and **24** were determined as flavan-3-ol and derivatives. Peak **12** and **18** were characterized as (−)-epicatechin and (+)-catechin, respectively. Both showed a [M − H]^−^ at *m*/*z* 289 and a fragment ion at *m*/*z* 245 corresponding to the loss of CO_2_ and they were confirmed by comparison with the retention time of standard. The deprotonated ions (peaks **22** and **24**) at *m*/*z* 441 produced the MS^2^ fragment ions at *m*/*z* 289, 169 and 125 corresponding to the deprotonated ion of (epi)catechin, gallic acid and decarboxylación of galloyl group, respectively. On the basis of mass spectral data and previously published data [30,31], this compound was identified as (epi)catechin gallate.

Peaks **2**, **4**–**11**, **13**–**17**, **19**–**21**, **23** and **25** were identified as proanthocyanidins and derivatives.

B-type proanthocyanidins were, qualitatively, the most abundant compounds in this extract [24,32]. Chemical structure of these compounds is based on the presence of (epi)catechin units which are linked by a single bond. Among these, eight procyanidin dimers (peaks **5**–**7**, **9**, **10**, **16**, **19** and **23**) with [M − H]^−^ ions at *m*/*z* 577 and four procyanidin trimers (peaks **2**, **4**, **8** and **15**) with [M−H]^−^ ions at *m*/*z* 865 were found. The major fragments were generated at *m*/*z* 451, after the neutral loss of 126 (C_6_H_6_O_3_, phloroglucinol) from the A ring of an (epi)catechin unit, at *m*/*z* 433 ([M-H-126-H_2_O]^−^), at *m*/*z* 425 corresponding to the loss of 152 (C_8_H_8_O_3_) which come from retro-Diels Alder (RDA) fission of the heterocyclic C ring and, sequentially, at *m*/*z* 407 ([M-H-152-H_2_O]^−^) and at *m*/*z* 289, due to the loss of 288 (C_15_H_12_O_6_, (epi)catechin−H_2_) by cleavages at the interflavanoid bonds [33].

Peaks **11**, **13**, **14**, **20**, **21** and **25** were detected at *m*/*z* 729 and tentatively assigned as galloyl(epi)catechin-(epi)catechin isomers [28]. These compounds produced the MS^2^ at *m*/*z* 577 (loss of a galloyl residue), at *m*/*z* 451 ([M-H-152-126]^−^), at *m*/*z* 433 ([M-H-152-126-H_2_O]^−^), at *m*/*z* 425 ([M-H-152-152]^−^), at *m/z* 289 (deprotonated (epi)catechin) and at *m*/*z* 169 (deprotonated gallic acid). One peak (**17**) was detected at *m/z* 743 and tentatively identified as a gallate of an A-type dimeric proanthocyanidin with (epi)catechin and (epi)gallocatechin subunits. This compound showed a MS^2^ base peak at *m/z* 591 corresponding to [M-H-152]^−^ [34].

Peaks **26**, **28**, **30**, **31** and **35** were identified as flavanols and derivatives. Peaks **26** and **31** had a [M − H]^−^ at *m*/*z* 463, and peak **28** and **30** at *m*/*z* 447. Product ion spectra of these peaks showed a major fragment ion at *m*/*z* 300. This could be due to the formation of the quinone anion (radical anion), which was obtained after homolytic cleavage of the *O*-glycosidic bond yielding the fragment at *m/z* 300.027, and it has been proposed as diagnostic for quercetin glycosides [35]. On the basis of mass spectral data and previously published data, these compounds were tentatively identified as quercetin hexoside (peak **26** and **31**), quercetin rhamnoside (peak **28**) and quercetin glucuronide (peak **30**) [36,37]. Peak **35** (*m/z* 301) was characterized as quercetin and confirmed by comparison with the retention time of the standard.

Peaks **29**, **33** and **36** were identified as flavanones and derivatives. Peak **36** (*m/z* 275) was tentatively identified as phloretin. Peaks **29** and **33**, with [M − H]^−^ at *m/z* 567 and 435, respectively, showed a MS^2^ fragmentation ion at 273 corresponding to phloretin moiety. This compound and its derivatives, mainly its glucoside, phorizin, are abundantly present in apples [38], especially in the peel [39] and seed [40]. Phloretin has been shown to inhibit of protein kinase C, human leukemia cell growth and bladder cancer and rat mammary adenocarcinoma cell growth [41].

#### 2.1.3. Other Compounds

Peak **1**, at *m*/*z* 341, was tentatively identified as sucrose according to its mass spectra and isotopic distribution as previously reported [42]. Peak **27**, with [M − H]^−^ ion at *m*/*z* 523, produced a major fragment at *m*/*z* 361 (loss of glucose). According to the literature, it was characterized as secoisolariciresinol glucoside [43,44]. Peak **32** (*m*/*z* 439), which was tentatively identified as amurenisin, has been reported in seeds of *Vitis amurensis* [45].

### 2.2. Quantification of Grape Seed Extract by HPLC-ESI-QTOF-MS

The sensitivity of the method was studied by defining the limits of detection (LOD) and quantification (LOQ) for individual compounds in standards solutions. The MS detection, based on the extracted ion chromatogram (EIC), was used to measure the peak areas. The EIC for each analyte was chosen regarding the measured [M − H]^−^ (Table 1). Table 2 summarizes the analytical parameters for the different compounds present in the grape seed extract. In order to quantify the amount of each compound, six calibration curves were prepared with the six standards commercially available: procyanidin B2, (+)-catechin, (−)-epicatechin, gallic acid, quercetin and quercetin-3-rutinoside. Compounds without a commercial standard available were quantitated using the calibration curve corresponding to the compound with the most similar structure. Oligomeric procyanidins, catechin derivatives, ellagic acid and quercetin derivatives were quantified with procyanidin B2, (+)-catechin, gallic acid and quercetin-3-rutinoside, respectively. Calibration curves were obtained for each standard with a good linearity (*R*^2^ > 0.99) by plotting the standard concentration as a function of the peak area obtained from HPLC-ESI-QTOF-MS analyses. The concentration ranges are also stated in Table 2, including the LOD and LOQ, which were calculated according to IUPAC recommendation [46].

The concentration of the extract was set at 2.5 g/L in all cases in order to fix in the considering working ranges. Three replicates of the extract were carried out and the results, expressed in μg/g (m/m, analyte/dry weighted extract), are also summarized in Table 2.

**Figure 3:**
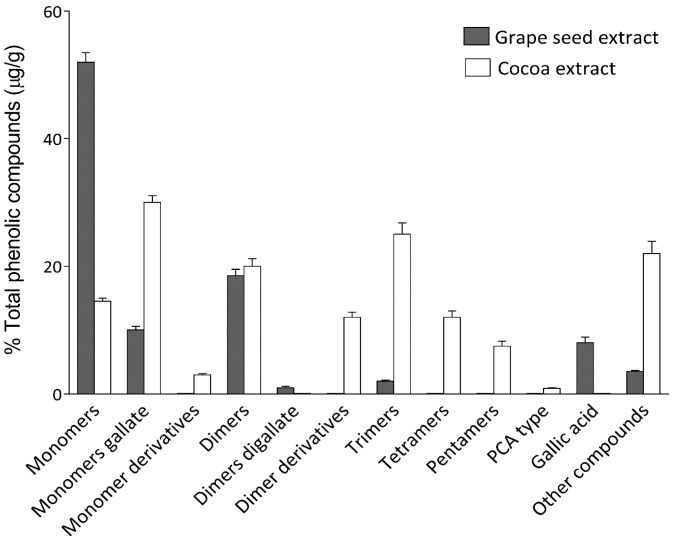
Comparative quantification of main compounds found in cacao and grape seed extracts. Quantitation values are expressed as μg of analyte per gram of dry extract.

As reported previously [27], cocoa extract shows higher levels of procyanidin oligomers such as tetramers, pentamers and hexamers than grape seed extract. In terms of dimer proanthocyanidins (B-type procyanidins and gallate derivatives), no differences have been observed. However, concerning monomeric flavan-3-ols, such as catechin or epicatechin, grape seed extract presents the highest proportion (Figure 3).

### 2.3. Total Phenolic and Flavan-3-ol Contents and in Vitro Antioxidant Activities Grape Seed Extract

As a previous step to the measurement of the antioxidant activity, the total phenolic and flavan-3-ol contents of the grape seed and cocoa extracts were quantified using the Folin–Ciocalteu method and Vanillin assay, respectively. Since these methods have a weak accuracy, they are widely used as an approximate method to semiquantitate phenolic compounds in plant extracts. The obtained values for each assay are shown in Table 3. On the basis of the dry weight, the total phenolic content in grape seed extract was 964 ± 82 mg GAE g^−1^ and total flavan-3-ol content was 988 ± 124 mg CE g^−1^. For cocoa extract, the results were 758 ± 82 mg GAE g^−1^ and 724 ± 121 mg CE g^−1^ for Folin–Ciocalteu and Vanillin assays, respectively.

Different in vitro methods were carried out in order to determinate the antioxidant activity of cocoa and grape seed extracts. These properties are primarily due to flavonoids, which can perform scavenging action on free radicals, metal chelating properties, reduction of hydroperoxide formation and their effects on cell signaling pathways and gene expression. The presence of the functional group “–OH” in the structure and its position on the ring of the flavonoid molecule determine the antioxidant capacity. Addition of “–OH” groups to the flavonoid nucleus will enhance the antioxidant activity, while substitution by –OCH_3_ groups diminishes the antioxidant activity. The antioxidant capacity of procyanidins is, in part, governed by the degree of polymerization. Grape seeds, which have a bigger content than skin and flesh on high-degree of polymerization procyanidins, show, therefore, the highest antioxidant power [47,48].

Trolox equivalent antioxidant capacity (TEAC) and ferric reducing antioxidant power (FRAP) are based on single-electron transfer mechanism. TEAC and FRAP are extensively used to establish the antioxidant activity in food [49] and biological samples [50], respectively. The oxygen radical absorbance capacity (ORAC) assay is performed in order to test the capacity of the extracts to quench peroxyl radicals, i.e., the hydrogen atom transfer ability. ORAC determination has become one of the most widely accepted methods to measure the antioxidant capacity of food, botanical, and biological samples [47].

Table 3 lists the antioxidant capacities of cocoa and grape seed extracts by TEAC, ORAC and FRAP methods. Accordingly, in TEAC and FRAP assays (single-electron transfer-based methods), the values for the grape seed extract were 6.1 ± 0.8 mmol of Trolox equivalents (TE) g^−1^ and 6.5 ± 0.5 mmol of Fe^2+^ equivalents (FE) g^−1^, respectively. Lower values are found for cocoa extract (4.2 ± 0.1 mmol TE g^−1^ and 4.0 ± 0.2 mmol FE g^−1^, respectively). Concerning ORAC assay, the values were 8.6 ± 0.7 and 7.0 ± 0.5 mmol TE g^−1^ for grape seed and cocoa extracts, respectively.

The comparison of these results with those of previous researchers is untenable due to differences in the nature of the sample and pre-concentration technologies, extraction systems, and assay methodologies.

By comparing all of our assays (which were made in parallel under the same conditions), grape seed extract showed high values of antioxidant activities and total phenolic and flavan-3-ol contents than cocoa extract. This could be a result of the higher content in flavan-3-ol, mainly the oligomeric forms, as well as the higher content in gallic acid in grape seed extract.

### 2.4. Anti-Inflammatory Activity of Grape Seed and Cocoa Extracts in HUVEC

**Figure 4:**
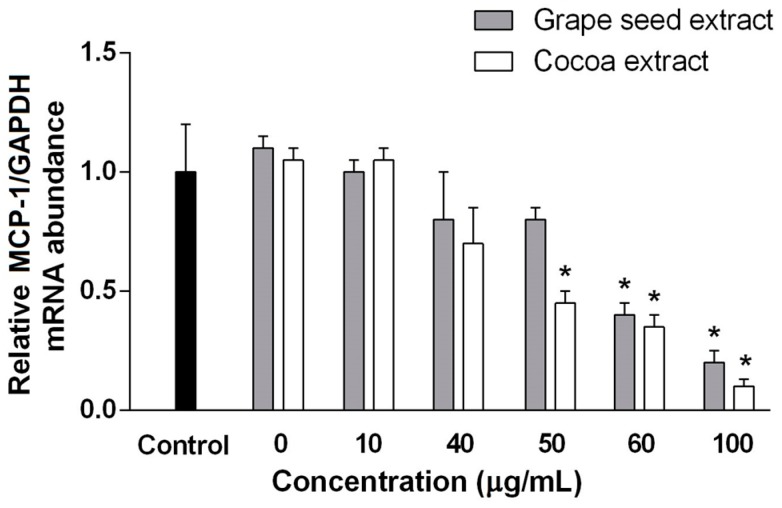
Effect of grape seed and cocoa extracts on production of relative MCP-1 mRNA levels in HUVEC. mRNA levels of MCP-1 were normalized using mRNA levels of glyceraldehyde 3-phosphate dehydrogenase (GAPDH). * statistically significant (*p*-value < 0.05).

As expected, the mRNA expression of MCP-1 (a proinflammatory cytokine) is decreased when a concentration more than 50 and 60 mg/mL of cocoa and grape seed extracts, respectively, are used (Figure 4). As observed, cocoa extract has a better response to inflammatory scenarios than grape seed extract. Although antioxidant and anti-inflammatory activities are generally related, we found that, in our case, the anti-inflammatory properties of proanthocyanins are not proportional to the observed antioxidant activity. The fact that polymeric proanthocyanins were found in cocoa extract but not in grape seed extract, could explain this effect, since the anti-inflammatory potential of proanthocyanins with high-degree of polymerization inhibits NF-κB activation and the secretion of eicosanoids and pro-inflammatory cytokines [51]. These results also agree with the activation of IL-4 secretion (an anti-inflammatory cytokine) [52] and the decreased levels on IL-8 (a pro-inflammatory cytokine) [53] by proanthocyanins with a high degree of polymerization.

## 3. Experimental

### 3.1. Chemicals

All chemicals were of HPLC-MS grade and used as received. Acetic acid and methanol for UHPLC were purchased from Fluka (Sigma-Aldrich, Steinheim, Germany) and Lab-Scan (Gliwice, Sowinskiego, Poland), respectively. Dimethyl sulfoxide (DMSO) was provided from Panreac (Barcelona, Spain). Milli-Q system from Millipore (Bedford, MA, USA) was used to obtain purified water was purified.

The standards, for the calibration curves, procyanidin B2, (+)-catechin, (−)-epicatechin, gallic acid (GA), quercetin and quercetin-3-rutinoside were purchase either from Fluka (Sigma-Aldrich, Steinheim, Germany) or Extrasynthese (Genay, France).

To measure the antioxidant capacity and total phenolic/flavanol-3-ol content, the following reagents were provided from the indicated suppliers: AAPH (2,2′-azobis-2-methyl-propanimidamide, dihydrochloride), ABTS [2,2′-azinobis (3-ethylbenzothiazoline-6-sulphonate)], ferric sulfate, fluorescein, Folin–Ciocalteu reagent, potassium persulfate, TPTZ (2,4,6-tripyridyl-*S*-triazine), Trolox (6-hydroxy-2,5,7,8-tetramethylchroman-2-carboxylic acid), vanillin and (+)-catechin from Sigma-Aldrich (St. Louis, MO, USA). From Panreac (Barcelona, Spain), gallic acid, dehydrated sodium phosphate, trihydrated sodium acetate, sodium acetate, ferric chloride, hydrochloric acid and sodium carbonate was purchased.

### 3.2. Sample Preparation

*V. vinifera* seed and *T. cacao* extracts were used in this study (Nutrafur, Elche, Spain). For polyphenols extraction, 10 mg of extract was dissolved in 1 mL of DMSO, then sonicated for 5 min, vortexed for 1 min, centrifuged for 5 min at 14,000 rpm and filtered through a 0.25 mm filter before the HPLC analysis.

### 3.3. Instrumentation

Analytical characterization of grape seed extract was performed using an Agilent 1200 series rapid-resolution LC system (Agilent Technologies, Palo Alto, CA, USA) equipped with a binary pump and an autosampler. The HPLC system was coupled to a quadrupole time-of-flight mass spectrometer (QTOF) mass spectrometer (Bruker Daltonics, Bremen, Germany) equipped with an electrospray ionization (ESI) interface (model G1607A from Agilent Technologies, Palo Alto, CA, USA). Fluorescence (ORAC) and absorbance (Folin–Ciocalteu, Vanillin assay, FRAP and TEAC) measures were carried out on a Synergy Mx Monochromator-Based Multi-Mode Micro plate reader (Bio-Tek Instruments Inc., Winooski, VT, USA) using 96-well polystyrene microplates.

### 3.4. Chromatographic Conditions

The compounds from grape seeds were separated at room temperature using a Zorbax Eclipse Plus C18 column (1.8 μm, 150 × 4.6 mm). The mobile phases were acetic acid 0.5% (solvent A) and methanol (solvent B). This multi-step linear gradient was applied: 0 min, 0% B; 5 min, 25% B; 15 min, 35% B; 20 min, 39% B; 38 min, 60% B; 40 min, 70% B; 42 min, 80% B; 44 min, 100% B; 46 min, 0% B; 48 min, 0% B. The initial conditions were maintained for 10 min. The injection volume was 10 μL. The flow rate used was set at 0.3 mL/min.

### 3.5. ESI-QTOF-MS Detection

The HPLC analysis were performed on a QTOF mass spectrometer equipped with an ESI interface. In negative ion mode, the capillary voltage operated at +3.5 kV. The other parameters of the source were set as follows: drying gas temperature, 220 °C; drying gas flow, 9 L/min; and nebulizing gas pressure, 2.5 bar. The detection was performed considering a mass range of 50–1200 *m*/*z*.

Molecular formulae for each analyte were proposed using the measured [M − H]^−^ ion and processed through the software DataAnalysis 4.0 (Bruker Daltonics), with an accepted accuracy threshold for confirmation of elemental compositions established at 5 ppm [54].

A 74900-00-05 Cole Palmer syringe pump (Vernon Hills, IL, USA) was used, during the development of the HPLC method, as an external instrument calibration directly connected to the interface, with a sodium acetate cluster solution. The calibration solution was injected at the beginning of each run and all the spectra were calibrated prior to the compound identification.

### 3.6. Total Phenolic and Flavan-3-ol Contents (TPC and TFC)

The TPC was measured by the Folin–Ciocalteu method with some modifications [45,55]. Cocoa and grape seed extracts were dissolved in methanol (different concentrations were tested). Phenol content was calculated based on the calibration curves of GA equivalents and expressed as mg GAE/g of dry matter. Measurements were made in triplicate.

Extract were analyzed for its TFC using a method described by Makkar and Becker (1993) [56], with some modifications [57]. Flavan-3-ol content was calculated based on the calibration curves of (+)-catechin equivalents and expressed as mg CE/g of dry matter. Measurements were made in triplicate.

### 3.7. Antioxidant Capacity Assays

The reduction of the radical cation of 2,2′-azinobis-(3-ethylbenzothiazoline-6-sulphonate) (ABTS) was performed by TEAC assay using a previously described method [58]. TEAC values were calculated using Trolox as a standard and reading absorbance at 734 nm in a microplate reader. The FRAP assay was carried out following the method described by Benzie and Strain (1996) [50]. FRAP values were calculated measuring the absorbance at 593 nm in a microplate reader and using FeSO_4_·7H_2_O as standard. To assay the capacity of the extracts to scavenge peroxyl radicals, a validated ORAC method was used [59] with some modifications [58] and measuring the excitation and emission wavelengths at 485 and 520 nm, respectively. A regression equation between the Trolox concentration and the net area of the fluorescence decay curve was used in order to obtain the final ORAC values. In all the antioxidant capacity assays, measurements were made in triplicate.

### 3.8. Anti-Inflammatory Activity Measurement

Anti-inflammatory experiments with HUVECs followed the procedures approved by our ethics committee, and were made as in Cádiz-Gurrea et al. (2013) [60]. Briefly, after cells reach confluence, they were treated with preconditioning medium containing TNF-α (R&D Systems, MN, USA) (5 ng/mL) for 6 h and then incubated for 48 h with fresh medium containing TNF-α 0.2 ng/mL and different concentrations (10–100 μg/mL) of grape seed and cocoa extracts.

After incubation, the supernatants were collected for ELISA analysis (RNeasy Mini Kit, Qiagen, CA, USA) and high-capacity cDNA reverse transcription kit (Applied Biosystems, CA, USA) was used to obtain cDNA using the primers for MCP-1 5′-ATGAAAGTCTCTGCCGCC-3′ and 5′-TTGCTTGTCCAGGTGGTC-3′ and for glyceraldehyde-3-phosphate-dehydrogenase (GAPDH) 5′-TCATTGACCTCAACTACATG-3′ and 5′-CAAAGTTGTCATGGATGACC-3′.

## 4. Conclusions

In the present work, HPLC-ESI-QTOF-MS has been confirmed to be a powerful analytical technique for separating and detecting phenolic and other polar compounds in a concentrated grape seed extract. With this methodology, 36 compounds were tentatively identified on the basis of their chromatographic retention, MS data, and MS/MS fragmentation pattern, and 30 compounds of them have been quantified. The most representative groups of compounds tentatively identified and quantified were proanthocyanidins (mainly monomers, dimers and galloyl derivatives). Besides these compounds, phloretin and its derivatives have been tentatively identified for the first time in *V. vinifera* seeds. These compounds have been reported to show several activities against diseases, i.e., antitumor effects.

Grape seed and cocoa extracts possess a significant antioxidant capacity to reduce peroxyl radicals by ORAC assay. Moreover, grape seed extract shows a stronger capacity to donate electrons by FRAP and TEAC assays, and a higher phenolic and flavan-3-ol contents. Finally, cocoa extract seems to have a better potential decreasing the expression of MCP-1, and therefore to prevent inflammation than grape seed extract due to its content on proanthocyanidins with high-degree of polymerization.

This work provides a better understanding of industrial byproduct from the winemaking process such as seeds. The importance of knowledge concerning this byproduct composition and activities is increasing due to its cheap source for the extraction of antioxidant compounds.

## Figures and Tables

**Table 1:** Retention time and mass spectral data of the compounds characterized in grape seed extract by HPLC-ESI-QTOF-MS and MS/MS in negative mode.

Peak	Proposed Compound	RT (min)	[M − H]^−^ Measured	[M − H]^−^ Calculated	Error (ppm)	mSigma	Fragmentation Pattern	Molecular Formula
1	Sucrose	5.5	341.108	341.109	2.7	18.0	Not fragmented	C_12_H_22_O_11_
2	Procyanidin C (isomer 1)	11.6	865.199	865.198	0.9	42.5	577.114; 289.076	C_45_H_38_O_18_
3	Gallic acid	12.6	169.013	169.014	6.7	3.8	125.024	C_7_H_6_O_5_
4	Procyanidin C (isomer 2)	14	865.197	865.198	1.4	20.8	577.134; 432.093	C_45_H_38_O_18_
5	Procyanidin B (isomer 1)	14.4	577.136	577.135	2.2	46.1	451.124; 289.076	C_30_H_26_O_12_
6	Procyanidin B (isomer 2)	14.6	577.136	577.135	1.3	53.1	425.075; 289.074	C_30_H_26_O_12_
7	Procyanidin B (isomer 3)	15	577.133	577.135	3.6	38.0	289.076	C_30_H_26_O_12_
8	Procyanidin C (isomer 3)	15.6	865.198	865.198	1.0	18.1	577.114; 451.123; 433.072; 289.065	C_45_H_38_O_18_
9	Procyanidin B (isomer 4)	16.4	577.136	577.135	1.9	48.5	425.088; 289.074	C_30_H_26_O_12_
10	Procyanidin B (isomer 5)	17.1	577.133	577.135	4.0	41.7	425.087; 289.073	C_30_H_26_O12
11	Galloyl(epi)catechin-(epi)catechin (isomer 1)	17.5	729.146	729.146	0.3	35.7	577.121; 289.074; 169.015	C_37_H_30_O_16_
12	(-)-Epicatechin	18.2	289.072	289.072	2.5	24.5	245.083	C_15_H_14_O_6_
13	Galloyl(epi)catechin-(epi)catechin (isomer 2)	18.7	729.148	729.146	2.5	54.4	577.132; 432.094	C_37_H_30_O_16_
14	Galloyl(epi)catechin-(epi)catechin (isomer 3)	19.2	729.147	729.146	1.0	50.7	577.131; 432.094; 169.014	C_37_H_30_O_16_
15	Procyanidin C (isomer 4)	19.8	865.200	865.198	1.8	22.3	432.094	C_45_H_38_O_18_
16	Procyanidin B (isomer 6)	20.3	577.134	577.135	1.8	49.2	432.092; 289.070	C_30_H_26_O_12_
17	Galloyl(epi)catechin-(epi)gallocatechin	20.9	743.125	743.125	0.0	28.7	591.170	C_37_H_28_O_17_
18	(+)-Catechin	23.1	289.072	289.072	3.4	23.1	245.083	C_15_H_14_O_6_
19	Procyanidin B (isomer 7)	24.3	577.133	577.135	4.4	16.5	407.076; 289.075; 245.044; 125.025	C_30_H_26_O_12_
20	Galloyl(epi)catechin-(epi)catechin (isomer 4)	25.2	729.144	729.146	2.1	19.6	577.131; 451.122	C_37_H_30_O_16_
21	Galloyl(epi)catechin-(epi)catechin (isomer 5)	25.4	729.144	729.146	3.2	14.1	577.131; 289.072	C_37_H_30_O_16_
22	(Epi)catechin gallate (isomer 1)	26.7	441.084	441.083	2.2	25.4	289.072; 169.015; 125.025	C_22_H_18_O_10_
23	Procyanidin B (isomer 8)	28	577.134	577.135	1.3	45.4	425.088; 289.073; 125.025	C_30_H_26_O_12_
24	(Epi)catechin gallate (isomer 2)	28.6	441.082	441.083	1.5	13.6	289.073; 169.015; 125.025	C_22_H_18_O_10_
25	Galloyl(epi)catechin-(epi)catechin (isomer 6)	30.6	729.145	729.146	0.9	35.2	577.117; 407.079; 289.071; 125.023	C_37_H_30_O_16_
26	Quercetin hexoside (isomer 1)	32.6	463.086	463.088	3.9	29.5	300.023	C_21_H_20_O_12_
27	Secoisolariciresinol glucoside	33.7	523.217	523.219	3.1	5.1	361.180	C_26_H_36_O_11_
28	Quercetin rhamnoside	34.9	447.034	447.093	0.3	31.2	300.028	C_21_H_20_O_11_
29	Phloretin xyloglucoside	35.5	567.169	567.172	4.9	33.1	273.073	C_26_H_32_O_14_
30	Quercetin glucuronide	36.1	477.067	477.067	1.5	7.9	300.028	C_21_H_18_O_13_
31	Quercetin hexoside (isomer 2)	36.7	463.071	463.067	1.7	10.5	300.027	C_21_H_20_O_12_
32	Amurenisin	37.1	439.066	439.067	3.1	5.3	Not fragmented	C_22_H_16_O_10_
33	Phloretin glucoside	38.4	435.129	435.130	0.6	7.2	273.072	C_21_H_24_O_10_
34	Ellagic acid	39	301.000	300.999	4.3	25.9	Not fragmented	C_14_H_6_O_8_
35	Quercetin	46	301.036	301.035	1.5	11.8	Not fragmented	C_15_H_10_O_7_
36	Phloretin	47.2	273.077	273.077	0.1	37.0	Not fragmented	C_15_H_14_O_5_

**Table 2:** Quantification data of compounds from grape seed extract: limit of detection (LOD) and quantification (LOQ), calibration range used for each calibration curve, linear equations and the coefficient of variation (R^2^). LOD and LOQ values were calculated for the available standards solely. Quantitation values are expressed as mean ± standard deviation (in μg of analyte per gram of dry extract).

Analyte	LOD (μg/mL)	LOQ (µg/mL)	Calibration Range (µg/mL)	Calibration Equations	*R* ^2^	Quantification (μg/g)
Procyanidin C (isomer 1)	–	–	0.39–6.25	*y* = 3 × 10^6^*x* – 3.6 × 10^4^	0.9945	117 ± 2
Gallic acid	0.254	0.848	0.5–12.5	*y* = 3.6 × 10^5^*x* + 2 × 10^4^	0.991	2491 ± 118
Procyanidin C (isomer 2)	–	–	0.39–6.25	*y* = 3 × 10^6^*x* – 3.6 × 10^4^	0.9945	79 ± 18
Procyanidin B (isomer 1)	0.096	0.321	0.39–6.25	*y* = 3 × 10^6^*x* – 3.6 × 10^4^	0.9945	143 ± 15
Procyanidin B (isomer 2)	–	–	0.39–6.25	*y* = 3 × 10^6^*x* – 3.6 × 10^4^	0.9945	474 ± 41
Procyanidin B (isomer 3)	–	–	0.39–6.25	*y* = 3 × 10^6^*x* – 3.6 × 10^4^	0.9945	2360 ± 296
Procyanidin C (isomer 3)	–	–	0.39–6.25	*y* = 3 × 10^6^*x* – 3.6 × 10^4^	0.9945	89 ± 14
Procyanidin B (isomer 4)	–	–	0.39–6.25	*y* = 3 × 10^6^*x* – 3.6 × 10^4^	0.9945	1623 ± 163
Procyanidin B (isomer 5)	–	–	0.39–6.25	*y* = 3 × 10^6^*x* – 3.6 × 10^4^	0.9945	291 ± 17
Galloyl(epi)catechin-(epi)catechin (isomer 1)	–	–	0.39–6.25	*y* = 3 × 10^6^*x* – 3.6 × 10^4^	0.9945	223 ± 16
(−)-Epicatechin	0.198	0.660	0.25-12.5	*y* = 7.7 × 10^5^*x* + 3.3 × 10^4^	0.9991	8900 ± 441
Galloyl(epi)catechin-(epi)catechin (isomer 2)	–	–	0.39–6.25	*y* = 3 × 10^6^*x* – 3.6 × 10^4^	0.9945	129 ± 14
Galloyl(epi)catechin-(epi)catechin (isomer 3)	–	–	0.39–6.25	*y* = 3 × 10^6^*x* – 3.6 × 10^4^	0.9945	1639 ± 156
Procyanidin C (isomer 4)	–	–	0.39–6.25	*y* = 3 × 10^6^*x* – 3.6 × 10^4^	0.9945	287 ± 43
Procyanidin B (isomer 6)	–	–	0.39–6.25	*y* = 3 × 10^6^*x* – 3.6 × 10^4^	0.9945	528 ± 79
Galloyl(epi)catechin-(epi)gallocatechin	–	–	0.39–6.25	*y* = 3 × 10^6^*x* – 3.6 × 10^4^	0.9945	164 ± 24
(+)-Catechin	0.207	0.688	0.25–12.5	*y* = 8.4 × 10^5^*x* + 1.6 × 10^5^	0.993	7747 ± 496
Procyanidin B (isomer 7)	–	–	0.39–6.25	*y* = 3 × 10^6^*x* – 3.6 × 10^4^	0.9945	122 ± 16
Galloyl(epi)catechin-(epi)catechin (isomer 4)	–	–	0.39–6.25	*y* = 3 × 10^6^*x* – 3.6 × 10^4^	0.9945	29 ± 4
Galloyl(epi)catechin-(epi)catechin (isomer 5)	–	–	0.39–6.25	*y* = 3 × 10^6^*x* – 3.6 × 10^4^	0.9945	22 ± 3
(Epi)catechin gallate (isomer 1)	–	–	0.25–12.5	*y* = 8.4 × 10^5^*x* + 1.6 × 10^5^	0.993	2744 ± 43
Procyanidin B (isomer 8)	–	–	0.39–6.25	*y* = 3 × 10^6^*x* – 3.6 × 10^4^	0.9945	274 ± 9
(Epi)catechin gallate (isomer 2)	–	–	0.25–12.5	*y* = 8.4 × 10^5^*x* + 1.6 × 10^5^	0.993	529 ± 26
Galloyl(epi)catechin-(epi)catechin (isomer 6)	–	–	0.39–6.25	*y* = 3 × 10^6^*x* – 3.6 × 10^4^	0.9945	84 ± 2
Quercetin hexoside (isomer 1)	–	–	0.25–6.25	*y* = 2 × 10^6^*x* – 3.8 × 10^4^	0.9983	97 ± 2
Quercetin rhamnoside	–	–	0.25–6.25	*y* = 2 × 10^6^*x* – 3.8 × 10^4^	0.9983	36 ± 2
Quercetin glucuronide	0.255	0.849	0.25–6.25	*y* = 2 × 10^6^*x* – 3.8 × 10^4^	0.9983	124 ± 12
Quercetin hexoside (isomer 2)	–	–	0.25–6.25	*y* = 2 × 10^6^*x* – 3.8 × 10^4^	0.9983	62 ± 2
Ellagic acid	–	–	0.5–12.5	*y* = 3.6 × 10^5^*x* + 2 × 10^4^	0.991	794 ± 86
Quercetin	0.207	0.690	0.25–10	*y* = 2 × 10^6^*x* – 2.4 × 10^5^	0.9992	59 ± 19

**Table 3:** Values for different antioxidant measurements performed with grape seed and cacao extracts. Values are expressed as mean ± SD.

Assays	Grape Seed	*T. cacao*
Folin-Ciocalteu ^a^	964.05 ± 82.29	758.33 ± 82.50
Vanillin assay ^b^	987.5 ± 123.7	723.6 ± 121.5
TEAC ^c^	6.1 ± 0.8	4.19 ± 0.14
FRAP ^d^	6.47 ± 0.47	3.95 ± 0.21
ORAC ^c^	8.62 ± 0.73	6.99 ± 0.5

^a^ Expressed in mg Gallic acid equivalents/g extract (dw); ^b^ Expressed in mg (+)-Catechin equivalents/g extract (dw); ^c^ Expressed in mmol Trolox equivalents/g extract (dw); and ^d^ Expressed in mmol FeSO_4_ equivalents/g extract (dw).
